# Experimental Voltammetry Analyzed Using Artificial Intelligence: Thermodynamics and Kinetics of the Dissociation of Acetic Acid in Aqueous Solution

**DOI:** 10.1021/acs.analchem.2c00110

**Published:** 2022-04-05

**Authors:** Haotian Chen, Danlei Li, Enno Kätelhön, Ruiyang Miao, Richard G. Compton

**Affiliations:** † Department of Chemistry, Physical and Theoretical Chemistry Laboratory, Oxford University, South Parks Road, Oxford OX1 3QZ, Great Britain; ‡ MHP Management- und IT-Beratung GmbH, Königsallee 49, 71638 Ludwigsburg, Germany

## Abstract

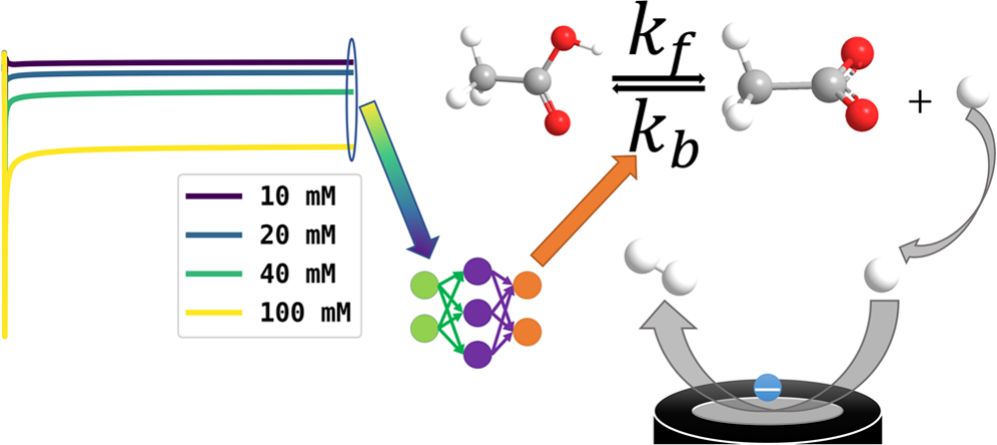

Artificial intelligence (AI) is used to quantitatively analyze the voltammetry of the reduction of acetic acid in aqueous solution generating thermodynamic and kinetic data. Specifically, the variation of the steady-state current for the reduction of protons at a platinum microelectrode as a function of the bulk concentration of acetic acid is recorded and analyzed giving data in close agreement with independent measurements, provided the AI is trained with accurate and precise knowledge of diffusion coefficients of acetic acid, acetate ions, and H^+^.

## Introduction

Machine learning has, despite its enormous potential, been used only sporadically in the quantification of analytes in different electroanalytical contexts and less in fundamental electrochemistry. For example, in food chemistry, a support vector machine approach was used to distinguish ale from lager based on cyclic voltammetry, and a neural network was able to estimate alcoholic content.^1^ In environmental chemistry, along with cyclic square-wave voltammetry, neural networks were applied to quantify the concentration of various pollutants in seawater, including copper, lead, mercury, paraquat (PQ), and Bisphenol-A (BPA).^2^ In biosensor development, by integrating with fast-scan cyclic voltammetry (FSCV) and an autoencoder, a variant of an artificial neural network, Mao et al. impressively achieved in vivo quantification of the concentration of dopamine, ascorbate, and NaCl in rat brains.^3^ Multicomponent detection of insulin and glucose in serum with the help of neural networks was also reported by Liu et al. recently.^4^ Beyond chemical analysis, in fundamental electrochemistry, Bond et al. reported the successful classification of electrode reaction mechanisms (specifically E-, EE-, and EC-type processes) using a convolutional neural network^5^ and claimed recognition of electrode kinetic between Butler–Volmer and Marcus–Hush types using Bayesian inference.^6^ More generally, at least in principle, when electrochemistry is equipped with machine learning for data analysis, the latter allows correlation and analysis of electrochemical data (including voltammograms and chronoamperograms) without the need for deploying mathematically analytical expressions.

Despite the emerging reports on the application to quantification of analytes, the study of electrochemical reactions and mechanisms with machine learning is still largely lacking. Bond and colleagues recently communicated the need for quantification of confidence limits and errors in parameter estimation when making a comparison of experimental data and simulated data. They attributed such absences to possible computational limitations.^7^ In response to the desirability for machine learning of parameters in electrochemical reactions, we recently communicated the theoretical study of training neural networks on simulated voltammograms to infer rate/equilibrium constants from the voltammograms of a dissociative CE_rev_ reaction and the reverse process of predicting voltammograms from such constants without recourse to further simulation.^8^ The general scheme of the dissociative CE reaction is
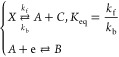
1where *k*_f_ and *k*_b_ are forward and reverse reaction rate constants, respectively, and the equilibrium constant, .

In this paper, we next develop this work in the context of experiment and apply the approach to the extraction of thermodynamic and kinetic parameters for the acetic acid dissociation reaction in aqueous solution enabling comparison with extensive independent reports in the literature so permitting verification and critical evaluation of the AI approach including limitations. In particular, authentic experimental data will contain finite background currents and possible contributions from migration and/or convection and/or double-layer charging, which inevitably distort, to a greater or lesser extent, the voltammetry from that predicted via simulation. Accordingly, it is important to address the question of whether the AI approach to the analysis of voltammetry can “live with” the inevitable imperfections of authentic experimental data.

Specifically, to facilitate proving the power of machine learning on parameter extraction from experiments, we quantitatively analyze current–concentration data to extract the thermodynamic and kinetic parameters (*K*_eq_ and *k*_f_) of acetic acid dissociation using machine learning. The predicted parameters are then checked via simulation and comparison of the results with experiments. Electro-reduction of acetic acid is known to follow a dissociative CE process^9,10^
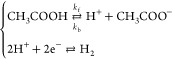
2

Table 1 shows the reported *K*_eq_ and *k*_f_ values at 298 K in different aqueous environments where the presence of the added electrolyte is known to cause both kinetic and thermodynamic salt effects, albeit relatively small in magnitude (see Table 1). *K*_eq_ values of acetic acid are readily measured, for example, most simply from the pH of acetic acid solutions and reported as 1.754 × 10^–5^ M in pure water.^11^ Higher values are seen when the electrolyte is present (i.e., 2.82 × 10^–5^ M in 0.1 M KCl solution^12^). Measurement of *k*_f_ has been made by a variety of methods.

**Table 1:** Literature Values of the Equilibrium and the Rate Constants of the Acetic Acid Dissociation Reaction

constant	solution composition	value and reference
***K***_**eq**_, M	CH_3_COOH in pure water	1.754 × 10^–5^,^11^
	CH_3_COOH, CH_3_COONa in pure water	1.743 × 10^–5^ to 1.784 × 10^–5^,^19^
	CH_3_COOH/0.1106 M KCl(aq)	2.805 × 10^–5^,^20^
	CH_3_COOH/0.1 M of KCl, NaCl or LiCl respectively	2.82 × 10^–5^, 2.79 × 10^5^, 2.85 × 10^–5^,^12^
	CH_3_COOH/1 M KCl(aq)	3.06 × 10^–5^,^21^
***k***_**f**_, **s**^–**1**^, nonelectrochemical methods	0.1 × 10^–3^ M CH_3_COOH in pure water	8 × 10^5^,^13^
	8.3 × 10^–5^ M CH_3_COOH in pure water	8.7 × 10^5^,^14^
	CH_3_COOH in pure water	1.91 × 10^5^,^15^
	CH_3_COOH coupled with bromocresol blue in pure water	1.3 × 10^6^,^16^
***k***_**f**_, **s**^–**1**^, electrochemical methods	2.7 × 10^–3^ M CH_3_COOH, 0.03 M CH_3_COONa/1 M KCl	3.46 × 10^6^,^9^
	2.17 × 10^–3^ M CH_3_COOH/1.475 M (CH_3_) _4_N(Cl)	1.58 × 10^6^,^10^
	20 × 10^–3^ M CH_3_COOH/0.1 and 0.3 M KCl	9.1 × 10^5^,^18^
	CH_3_COOH/1 M KCl(aq)	3 × 10^5^,^22^
	2.5 × 10^–3^ M CH_3_ COOH, 5 × 10^–3^ M CH_3_ COONa/50:50 water-ethanol. Ionic strength adjusted to 1 M with KCl	2.9 × 10^5^,^23^
	CH_3_COOH/LiCl(aq). Ionic strength adjusted to 1 M with LiCl	1.39 × 10^6^,^24,25^

Nonelectrochemical methods reported include the electrical pulse methods,^13^ the high field dispersion and temperature jump method,^14^ and the electric field jump (E-jump) relaxation technique.^15^ Although acetic acid does not absorb at visible wavelength, a colored acid–base indicator, Bromocresol green, was coupled in solution to enable spectroscopic detection using a square-wave field-effect apparatus.^16^

Electrochemical methods reported include voltammetry using a hydrodynamic modulated rotating disk electrode and analysis using the modified Koutecky–Levich equation,^9,17^ the polarography on acetate-acetic acid solution of low buffering capacity,^10^ and a two-cell technique, each with a rotating electrode connected by a Wheatstone bridge circuit.^18^ A table of individually measured *k*_f_ and *K*_eq_ values is shown in Table 1. The values of *k*_f_ range widely from 1.91 × 10^5^ to 3.46 × 10^6^ s^–1^ in different solution compositions.

In the following, we report a simple three-step electrochemical approach for estimating these two constants. This paper follows the work flow of method A reported before,^8^ but the technical implementation is bespoke to account for the exact experimental data.

First, measurements are made of the steady-state current for the reduction of protons as a function of the bulk acetic acid concentration *c*_CH_3_COOH,total_^*^ at a platinum microdisk electrode. This approach was preferred to cyclic voltammetry because the steady-state currents obtained at a microelectrode are independent of electrochemical rate constants and transfer coefficients, whereas understanding and simulating a full voltammogram either at a macroelectrode or a microelectrode for the reduction of H^+^ from acetic acid dissociation would require a confident knowledge of the mechanism and the kinetic parameters of the hydrogen evolution reaction on the Pt electrode, which remains controversial.^26,27^ Under the conditions employed in the present study, the steady-state current depends only on *k*_f_, *K*_eq_, and *c*_CH_3_COOH,total_^*^ assuming prior knowledge of diffusion coefficients.

Second, a simulation of the expected limiting currents with different *k*_f_, *K*_eq_, and bulk concentrations of acetic acids was conducted to obtain the steady-state current under different conditions and a neural network was trained and tested with simulated data. The features used for training were the steady-state currents at different bulk concentrations of acetic acid and targets were the *k*_f_ and *K*_eq_ values for the acetic acid CE process.

Third, steady-state currents obtained from experiments were fed into the trained network to give predictions of *k*_f_ and *K*_eq_ values, which were compared with those in Table 1 facilitating generic insights into the AI approach. More generally, the latter offers the prospect of simulation-free approaches to the quantitative analysis of voltammetric data in which simulations are only used to initially train the AI but thereafter there is no resort to expensive, commercial software, as the exact implementation of which can be user sensitive. The methodology thus promotes the analysis of data in a manner that is easily comparable between laboratories. To this end, we provide the simulation and machine learning programs for acetic acid reduction reported, along with raw experimental data, at https://github.com/nmerovingian/Acetic-Acid-Dissociation-AI. The simulation and machine learning programs for the theoretical study reported before^8^ can be found at https://github.com/nmerovingian/dissociativeCE-Simulation-MachineLearning. The resources provided will enable the users to fully reproduce the results reported and possibly further explore the application of AI using the training data provided.

## Theory

In this section, we first discuss the formal potential of the H^+^/H_2_ couple and the expected half-wave potential for the reduction of protons in the case that the proton reduction reaction is electrochemically reversible. Second, we outline the simulation of the expected transport-limited currents for the proton reduction as a function of *K*_eq_ and *k*_f_, noting that the computational approach is given in ref (8) apart from small changes in boundary conditions as outlined below.

### Formal Potential and Half-Wave Potential of the H^+^/H_2_ Couple

The formal potential, *E*_f,H^+^/H_2__^0^ of the H^+^/H_2_ couple has been shown to be given by
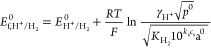
3where *E*_H^+^/H_2__^0^ is the standard electrochemical potential and γ_H^+^_ is the activity coefficient of H^+^ ion in solution; *k*_s_ and *c*_s_ are the salt parameter and salt concentration, respectively, which account for the salt-out effect in an electrolytic solution; *p*_0_ and *a*_0_ are standard pressure and standard activity, which are 1 bar and 1 M, respectively; *K*_H_2__ is Henry’s law constant for H_2_; and *R*, *T*, and *F* are the gas constant, temperature, and Faraday constant, respectively. Using parameters from Table 2, *E*_f,H^+^/H_2__^0^ is calculated as −0.341 V vs saturated calomel electrode (SCE).^28^

**Table 2:** Parameters Used for Calculation of the Formal Potential of the H^+^/H_2_ Redox Couple and for Simulation

parameter	explanation	value
*E*_H^+^/H_2__^0^ vs SCE	standard potential of H^+^/H_2_ vs saturated calomel electrode	–0.241 V,^30^
*D*_CH_3_COOH^–^_	diffusion coefficient of acetic acid	1.29 × 10^–9^ m^2^ s^–1^,^11^
*D*_CH_3_COO^–^_	diffusion coefficient of acetate	1.089 × 10^–9^ m^2^ s^–1^,^11^
*D*_H^+^_	diffusion coefficient of hydrogen ion	9.311 × 10^–9^ m^2^ s^–1^,^11^
*D*_H_2__	diffusion coefficient of hydrogen	5.11 × 10^–9^ m^2^ s^–1^,^11^
γ_H^+^_	activity coefficient of hydrogen ion	0.754,^31^
*K*_H_2__	Henry’s law constant of hydrogen	1292 bar M^–1^,^32^
*c*_KNO_3__	concentration of KNO_3_ electrolyte in experiment	0.1 M
*k*_KNO_3__	salt parameter of KNO_3_	0.07 M^–1^,^33^

The half-wave potential assuming an electrochemically reversible H^+^/H_2_ couple for the reduction of protons at a uniformly accessible electrode has been derived previously^28^
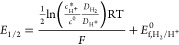
4where *E*_1/2_ is the half-wave potential and *c*_H^+^_^*^ and *c*^0^ are the bulk concentration of acetic acid and reference concentration (1 M), respectively. The dependence of the half-wave potential on the bulk proton concentration arises from the nonunity stoichiometry of the reaction.^28,29^ The formal potential *E*_f,H^+^/H_2__^0^ as derived above is −0.341 V vs SCE. Using the reported p*K*a value of 4.756 of acetic acid^11^ and bulk concentration of acetic acid as 10 mM, *c*_H^+^_^*^ is calculated to be 4.10 × 10^–4^ M and *E*_1/2_ is estimated as −0.448 V vs SCE. On the other hand, assuming *c*_H^+^_^*^ as the bulk concentration of acetic acid, the calculated *E*_1/2_ is −0.408 V vs SCE. Clearly, from the CE process of describing the acetic acid reduction with the hypothetical assumption of reversible electrochemistry, the half-wave potential would be expected to lie between −0.408 and −0.448 V.

### Simulation Equations

The mass transport is assumed to be exclusively diffusive, and the diffusion equations coupled with chemical reactions are solved to show how the steady-state limiting current at a microelectrode depends on the parameters *k*_f_ and *K*_eq_ for different acetic acid concentrations. Note that the electrochemical reaction on a microdisk electrode of radius r can be approximated as a hemispherical electrode with radius to reduce the diffusion problem from two dimensions to one dimension. The relevant steady-state diffusion equations can be formulated as
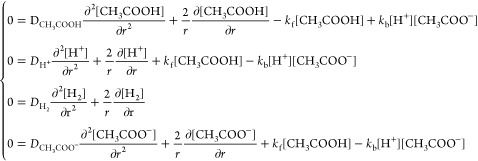
5where *D*_*j*_ is the diffusion coefficient for species *j*.

If *r*_e_ is the radius of the hemispherical electrode, the boundary conditions at the surface of the electrode for the steady-state reduction of protons are
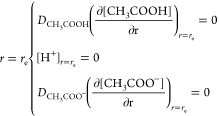
6

The boundary conditions for the outer boundary of simulation are
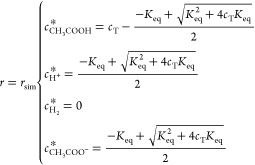
7where *c*_T_ = *c*_CH_3_COOH,total_ is the concentration of acetic acid added to the solution before chemical equilibrium forming proton and acetate, which are assumed absent in the initial solution. In the absence of electrolysis, *c*_CH_3_COOH,total_ = *c*_CH_3_COOH_^*^ + *c*_CH_3_COO^–^_^*^ and *r*_sim_ is the outer boundary of the simulation as discussed in ref (8).^8^

## Experimental Section

### Chemicals

Acetic acid (CH_3_COOH, 99.8%), potassium nitrate (KNO_3_, 99%), potassium chloride (KCl, 99%), and hexaammineruthenium (III) chloride (Ru(NH_3_)_6_Cl_3_, 98%) were purchased from Sigma-Aldrich (Dorset, UK) and used as received. All solutions were prepared with deionized water (of resistivity 18.2 MΩ cm at 298 K, Millipore).

### Voltammetry and Chronoamperometry of Acetic acid at a Pt Microdisk Electrode

All electrochemical measurements were performed with a μAutolab Type III potentiostat analyzer using a standard three-electrode arrangement in an optimized and thermostatted electrochemical cell^34^ in a Faraday cage. A Pt microdisk electrode (diameter: approximately 10 μm) was polished with three grades of successively finer aluminum powder (1.0, 0.3, and 0.05 μm), washed with deionized water, dried with N_2_ flow, and served as the working electrode. The counter and reference electrodes were a platinum wire and a saturated calomel electrode (SCE), respectively. The radius of the Pt microdisk electrode was calibrated electrochemically as 4.97 ± 0.05 μm by analyzing the steady-state voltammetry of 1.0 mM [Ru(NH_3_)_6_]^3+^ in 0.1 M KCl aqueous solution, using the reported diffusion coefficient for [Ru(NH_3_)_6_]^3+^ of 8.43 × 10^–10^ m^2^ s^–1^ at 298 K in 0.1 M KCl solution.^35^

**Figure 1:**
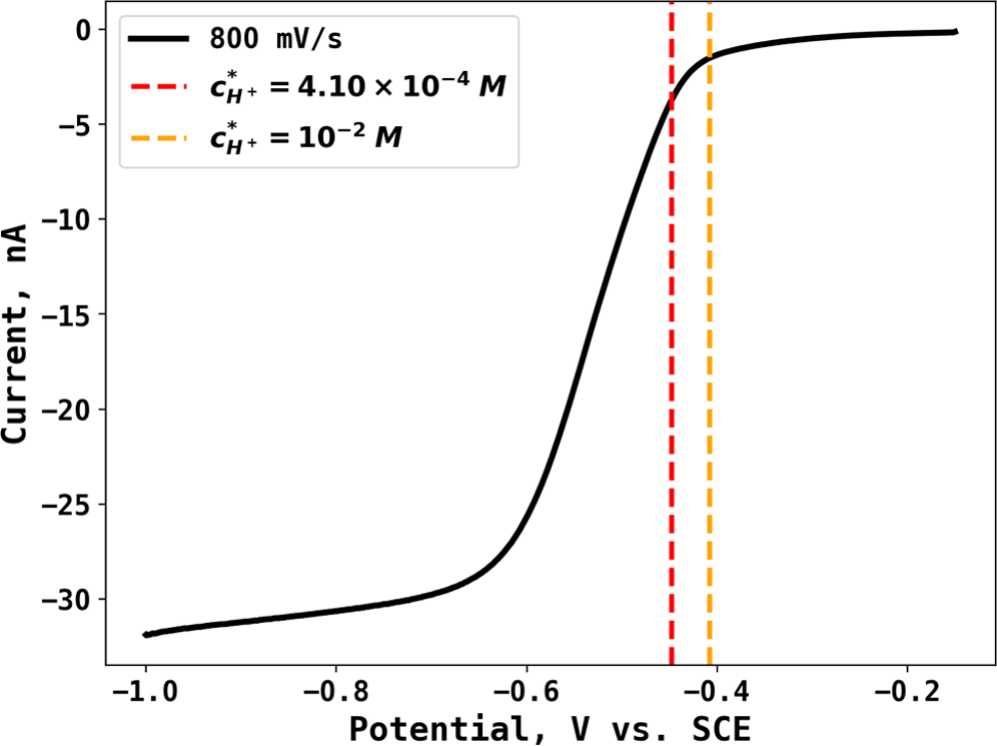
Linear sweep voltammetry of 10 mM acetic acid in 0.1 M KNO_3_ at a scan rate of 800 mV s^–1^ from −0.15 to −1.0 V vs SCE. Dashed lines show the calculated half-wave potentials at different bulk concentrations of H^+^.

Linear sweep voltammetry of 10 mM acetic acid in 0.1 M KNO_3_ was performed in the potential range of −0.15 to −1.0 V vs SCE at a scan rate of 800 mV s^–1^, as shown in Figure 1. The half-wave potential is measured at −0.570 V vs SCE, significantly smaller than the half-wave potential of the electrochemically reversible H^+^/H_2_ couple at different concentrations (−0.408 to −0.448 V), suggesting that the reduction of H^+^ on the *Pt* microdisk electrode was at least partly electrochemically irreversible.^28^

Acetic acid solutions with four concentrations (10, 20, 40, and 100 mM) were prepared in the 0.1 M KNO_3_ supporting electrolyte. The solution was degassed for 10 min with N_2_ before voltammetric or chronoamperometric measurements, and the temperature was stabilized at 298 ± 1 K via a digital temperature controller (SCT1 Digital contact thermometer).^34^ Current time transients for analysis via AI were recorded at an applied potential of −1.0 V vs SCE for a duration of 10 sec. The working electrode was polished with 0.05 μm aluminum powder and washed with deionized water between each experiment. Three repeated chronoamperometries were performed for each concentration.

## Simulation and Machine Learning

The simulation program was written in Python. Multiprocessing was used for parallel computing of the working surfaces on an Intel E5 processor. The nonlinear diffusion equations were solved using the finite difference method by discretizing the diffusion equations using both expanding space grid and time grid. The resulting multidiagonal matrix was solved using the Newton–Raphson method for at most 10 iterations.^36^ If the mean absolute error in dimensionless concentration was smaller than 10^–12^, additional iterations were skipped to save time without significant compromise of accuracy. The convergence of the simulation was checked, and the results can be found in Testing and Verification of the Simulations section in the Supporting Information.^37−39^

The working surfaces from simulations were used to train a multiheaded Dense Neural Network (DNN) written in TensorFlow.^40^ The DNN was trained by using the simulated steady-state currents at different concentrations as features to predict the corresponding log_10_*k*_f_ and log_10_*K*_eq_ as targets. Note that the targets were in logarithm form to reduce the exploding/diminishing gradient problem.^41^ The DNN has only 4 hidden layers with relatively small numbers of neurons (<100) to avoid overfitting: a smaller network forced itself to predict rate constants instead of “memorizing” them.

The implementation of simulation and machine learning programs along with scripts for visualization and feature engineering can be found at https://github.com/nmerovingian/Acetic-Acid-Dissociation-AI. Raw experimental data is also provided in the repository. The more general simulation and machine learning programs for dissociative CE reaction reported before^8^ can be found at https://github.com/nmerovingian/dissociativeCE-Simulation-MachineLearning. Note that because of the stochastic nature of the neural network, and different hardware and operating systems from our workstation, the users’ machine learning results may vary slightly from the authors’, but generally to less than 0.1 on the log10 scale for prediction of rate and equilibrium constants. The simulation results are expected to be consistent in a different computing environment.

## Results and Discussion

Figure 1 shows the voltammogram for which measurements were made. Current data was measured at a potential of −1.0 V vs SCE corresponding to the limiting current and the attainment of the steady-state current assessed by chronoamperometry as described in the next section. Specifically, the measurement of the limiting current as a function of the bulk concentration of acetic acid is used to avoid the need for any electrode kinetic data or assumption.

### Chronoamperometry of Acetic Acid

To identify the steady-state currents, current–time transients for four different concentrations of acetic acid between 10 and 100 mM in 0.1 M KNO_3_ were recorded at a Pt microdisk electrode by stepping the potential from a value where no current flowed to an applied potential of −1.0 V vs SCE, corresponding to a sufficiently negative potential for a diffusion-limited reduction of protons. The corresponding chronoamperograms at four concentrations are reported in the Chronoamperogram section in the Supporting Information, which showed that steady-state behavior is observed after 2 s. Erring on the side of caution, currents at *t* = 10 s were chosen for quantitative analysis, assumed to be steady state and measured as −30.5 ± 0.4, −55.2 ± 0.3, −102 ± 1, and −238 ± 4 nA for 10.0, 20.0, 40.0, and 100 mM of acetic acid with three separate chronoamperograms averaged for each concentration. The steady-state currents did not scale proportionally with the concentration of acetic acid since the reduction of acetic acid obeys the dissociative CE mechanism. The concentrations of acetic acid and the measured steady-state currents were utilized as experimental features for machine learning to predict rate and equilibrium constant as described below.

### Simulation of Steady-State Limiting Currents

**Figure 2:**
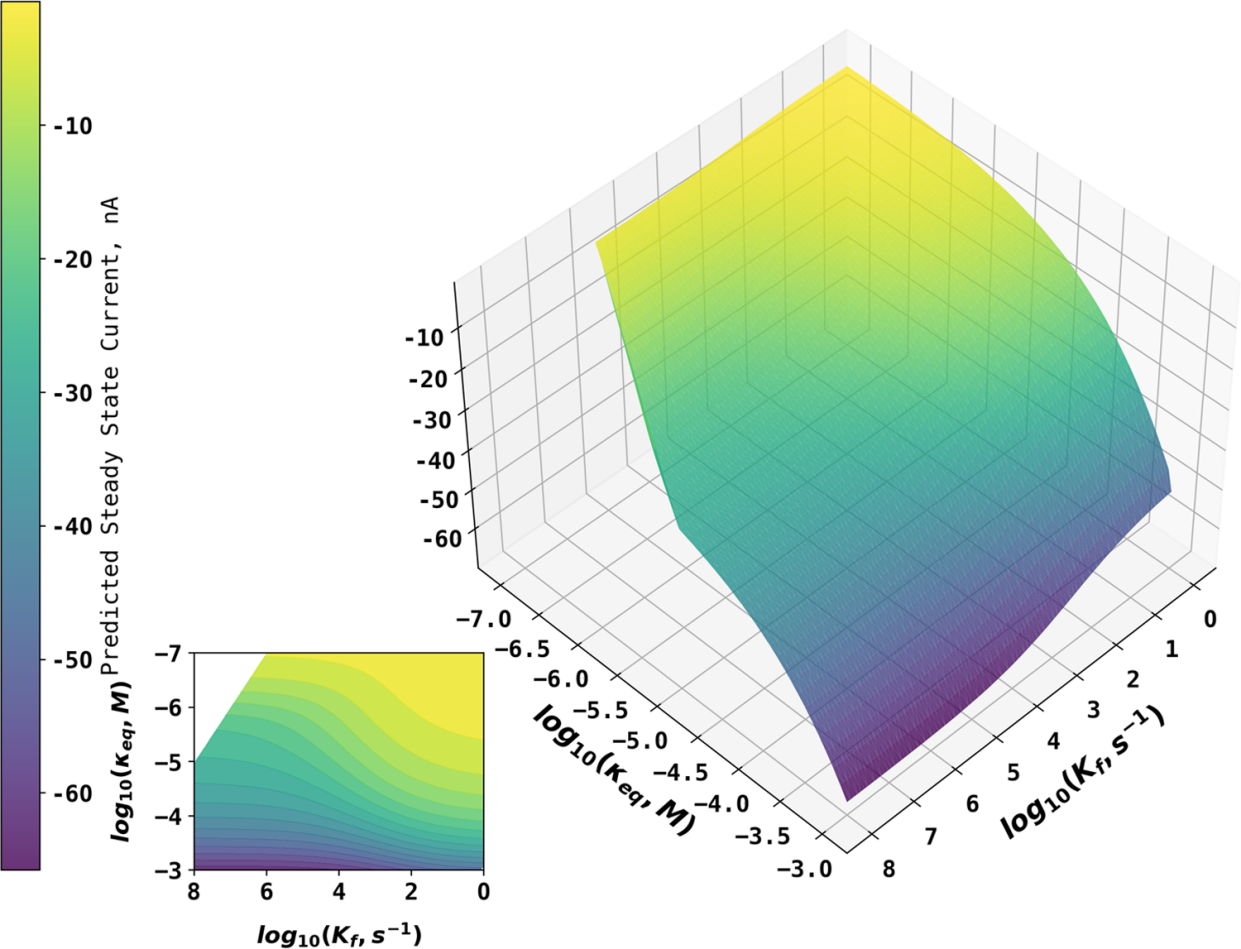
Working surface showing the steady-state currents at different *k*_f_ and *K*_eq_ values for a bulk concentration of acetic acid of 10 mM. Note: the apparent “kink” in the left of the surface is because it is not parallel to the log_10_*K*_eq_ axis but rather cross (*k*_f_ and *K*_eq_) space at an angle. The smooth continuity of the surface is emphasized by the contour plot.

A wide range of *k*_f_ (1–10^8^ s^–1^) and *K*_eq_ (10^–3^–10^–8^ M) values were applied to simulate the steady-state limiting currents at four different bulk concentrations of acetic acid (10, 20, 40, and 100 mM). The diffusion coefficients of all species reported in the literature are given in Table 2. Note that some combinations of *K*_eq_ and *k*_f_ values would generate *k*_b_ values exceeding a reasonable magnitude, so simulations were only performed if *k*_b_ ≤ 10^13^ M^–1^ s^–1^ with the latter values selected to give a considerable margin of error. The steady-state currents at different *k*_f_ and *K*_eq_ values are represented on the working surfaces such as the one shown in Figure 2 for a bulk concentration of acetic acid of 10 mM. Figure 2 shows that the steady-state current increases in magnitude with increasing *K*_eq_ and *k*_f_. Increasing *K*_eq_ can increase the magnitude of steady-state current since higher *K*_eq_ increases the bulk concentration of electroactive H^+^ at equilibrium. Higher *k*_f_ increases the extent of acetic acid dissociation on the voltammetric timescale to replenish H^+^ consumed during electrochemical reduction. The working surfaces for bulk concentrations of acetic acid of 20, 40, and 100 mM can be found in the Working Surfaces for the Steady-State Limiting Current section in the Supporting Information.

### Training and Testing the Neural Network

**Figure 3:**
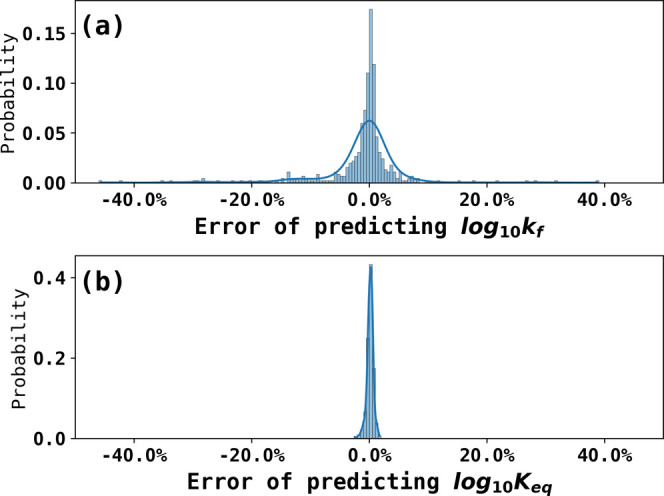
Error of predicting the rate and equilibrium constants from an independent testing dataset composed of simulated steady-state currents. (a) Errors of predicting log_10_*k*_f_ 90.5% predictions of log_10_*k*_f_ were within 10% errors and (b) errors of predicting log_10_*K*_eq_ 100% predictions of log_10_*K*_eq_ were within 5% errors.

Using the working surfaces, the neural network was trained to predict log_10_*k*_f_ and log_10_*K*_eq_ using steady-state currents at four different concentrations of acetic acid and trained for 2000 epochs. The loss function was the mean absolute error (MAE) and the optimizer was Adam (learning rate = 0.001).^42^ To evaluate the performance of the neural network model after training, it was tested with independent testing datasets and the results are shown in Figure 3, as 90.5% predictions of log_10_*k*_f_ were within 10% error and 100% predictions of log_10_*K*_eq_ were within 5% error. The lower accuracy of predictions of log_10_*k*_f_ compared to log_10_*K*_eq_ arises because log_10_*k*_f_ had a relatively smaller effect on the steady-state current but the network was judged sufficient for the prediction of constants from experimental results. The performance was benchmarked with a third-degree polynomial regression, which results in 61.2% prediction of log_10_*k*_f_ within 10% error and 86.7% prediction of log_10_*K*_eq_ within 5% error. Details are shown in the Benchmark section in the Supporting Information.

### Predicting Rate and Equilibrium Constants with Experimental Data

Using the steady-state current at the four different concentrations as the input of the neural network trained in the last step, the neural network predicted *k*_f_ and *K*_eq_ values in the logarithmic scale as 6.47 and −4.77, which convert to 2.95 × 10^6^ s^–1^ and 1.70 × 10^–5^ M. The 95% prediction intervals for the neural network were estimated using the bootstrap method.^43^ First, 500 observations were sampled from testing datasets and repeated 100 times to obtain 100 bootstrapped samples. Second, each bootstrap sample was trained in an independent neural network to obtain an empirical distribution of bootstrap predictions. At the 5% significance level, the upper and lower limits were, respectively, 97.5 and 2.5% quantile obtained from the bootstrap distribution. The 95% prediction intervals were calculated as 2.78 × 10^6^–3.13 × 10^6^ s^–1^ and 1.67 × 10^–5^–1.71 × 10^–5^ M for predicted *k*_f_ and *K*_eq_, respectively. These values are fully consistent with the accepted literature values shown in Table 1 showing the power of the proposed AI approach in extracting kinetic and thermodynamic data.

### Testing the Approach with Different Diffusion Coefficients

Not all systems under study will have as well-characterized diffusion coefficient as the acetic acid dissociation reaction described above. Sometimes, diffusion coefficients are unknown and frequently uncertain. It is therefore pertinent to ask how well the AI approach responds to variations in the diffusion coefficients used for the proton, acetic acid, and acetate ions.

To address this question, we compared the performance of the three-step electrochemical approach with five different sets of diffusion coefficients and the predicted constants using simulated steady-state currents, as shown in Table 3. The values of *k*_f_ and *K*_eq_ obtained for the wrong diffusion coefficients that deviate from the accepted values give noticeably wrong answers.

**Table 3:** Five Cases Considered When Varying Diffusion Coefficients of Species and the Predicted ***k***_**f**_ and ***K***_**eq**_ Values

case #	description	predicted *k*_f_, s^–1^	predicted *K*_eq_, M
1	diffusion coefficients shown in Table 2	2.95 × 10^6^	1.70 × 10^–5^
2	increasing diffusion coefficients in Table 2 by 10%	3.02 × 10^3^	5.13 × 10^–6^
3	decreasing diffusion coefficients in Table 2 by 10%	>10^10^	3.47 × 10^–6^
4	all diffusion coefficients set to 10^–9^ m^2^ s^–1^	9.05 × 10^2^	1.56 × 10^–3^
5	*D*_H^+^_ = 9.311 × 10^–9^ m^2^ s^–1^, other species 10^–9^ m^2^ s^–1^	>10^10^	1.07 × 10^–5^

In cases 2 and 3, the diffusion coefficients were arbitrarily set higher or lower than the accepted values by 10%. The effect is that for lowered diffusion coefficients, the *K*_eq_ and *k*_f_ values increase to provide more or more rapid dissociation, while for increased diffusion coefficients, the opposite occurs and the two parameters decrease to reduce the number of protons formed through dissociation on the voltammetric timescale. These trends are exactly what is expected since we are using the limiting current to probe the two parameters of interest and so the inferred values will be very sensitive to the rate of diffusion in addition to *k*_f_ and *K*_eq_. The steady-state concentration profile can be found in the Concentration Profile section in the Supporting Information.

It is interesting first that the values of *k*_f_ and *K*_eq_ are changed so markedly, highlighting a limitation of the electrochemical method for their measurement, and second that the AI approach stresses the requirement for the accurate parameter input in a way that is much more emphatic than using traditional curve-fitting approaches.

In the example chosen for study, all three diffusion coefficients are well known and the used ones in case 1 are considered to be reliable especially as they are measured via conductivity or Gouy interferometry.^44^ In some cases, where the diffusion coefficients are unknown, an experimenter might be tempted to estimate or guess the relevant parameters. Thus, in cases 4 and 5, we investigate this approach and fix some or all of the diffusion coefficients as 10^–9^ m^2^ s^–1^. This set of low values significantly underestimates the *D* of H^+^ and therefore leads to high *k*_f_ estimates. The steady-state concentration profiles of CH_3_COO^–^, H^+^, and CH_3_COOH in cases 1, 4, and 5 are shown in the Supporting information when (a) *c*_CH_3_COOH,total_^*^ = 100 mM and (b) *c*_CH_3_COOH,total_^*^ = 10 mM and are to be contrasted with those generated from more reliable diffusion coefficients.

The five cases discussed above showed that incorrect simulation parameters notably diffusion coefficients lead to incorrect input data for the neural network training and generate unreliable predictions. Thus, in practice, we recommend major caution with simulation parameters for accurate simulations and predictions.

## Conclusions

We have shown how artificial intelligence methods can be developed and trained to analyze electrochemical data and extract reliable kinetic and thermodynamic parameters, which compare well with those independently measured both by electrochemical and nonelectrochemical methods given a mechanism for the electrode reaction is known. The specific approach previously advocated^8^ has been shown to be effective when using simulation to train and validate AI programs and then applied to authentic experimental data. However, it is useful to assess the strengths and especially the limitations of the approach. We note that the method meets the challenge presented by Bond and colleagues of giving a well-defined and experimenter-independent method of data analysis. However, it must be recognized that we have applied it to a well-defined system where the mechanism of the electrode process under evaluation is known. This is required to perform the simulation necessary to train the AI. Moreover, because the chemistry is clear, it is possible to simplify the number of parameters needed for training by adopting the reliable literature data for the diffusion coefficients of the three species controlling the magnitude of the current, acetic acid, acetate ions, and protons. Even so, from the analysis using deliberately wrong diffusion coefficients, it is clear that even relatively small errors in the values lead to significant errors in the inferred *k*_f_ and *K*_eq_ values. That said, AI allows the sensitivity of the values to be readily checked and the inferences caveated in a way that is not always easily adopted in conventional analysis of voltammogram.

It is evident that the approach of using simulation to train the AI requires a clear understanding of the likely chemistry even if the experimenter is willing to simulate and train AI for different possible mechanisms. Overall, this points to a possible need to make complementary measurements, notably spectro-electrochemistry, and/or good chemical intuition to identify realistic chemistry. In the above, we deliberately selected to focus our analysis on the limiting current data from our experiments to make our conclusion independent of the mechanism and electrode kinetics of the H^+^/H_2_ redox couple, which would influence cyclic voltammetric data (peak current and peak potentials). The speculative application of a trained network for more complex chemistry is fraught with risk, possibly extreme risk. Thus, in the context of the dissociative CE mechanism, such a process undoubtedly underpins the oxidation of hydrazine in aqueous solution where deprotonation of N_2_H_5_^+^ prior to oxidation of N_2_H_4_ is required at some electrodes; however, the oxidation process is self-inhibiting since it produces nitrogen and protons^45,46^
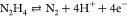
which act to change the pH of the solution local to the electrode. The application of an AI program trained for a simple dissociative CE process could not capture the essential content of the voltammetry/chronoamperometry and requires chemical expertise from the experimenter. Similarly, the voltammetry of blood reveals signals attributed to the electro-reduction of oxygen released from oxy-hemoglobin close to an electrode.^47−49^ Again, the chemistry is not simple: four oxygen molecules are bound to each hemoglobin and have different kinetics and thermodynamics of release. A chemically over-simplified analysis using AI trained for a simple mechanism would likely give erroneous or misleading output. Further, it is evident that the use of low-quality training data presents implantation risk and that this grows with the complexity of the mechanism because of the number of parameters required. Knowledge of accurate diffusion coefficients is one specific problem and, of course, is intrinsic to all electrochemical data analysis problems. We predict that the role of human intelligence (“HI”) will remain dominant over the artificial form for the foreseeable future in electrochemistry at least except for some niche applications. One such immediate niche lies in the analysis of electrode reaction mechanisms where the experimentalist is confident of the generic nature of the reaction, for example, CE, EC, ECE, etc. As we have shown, the AI approach allows for simulation-free data analysis and hence makes the extraction of parameters independent of the experimenter so better facilitating interlaboratory comparisons.

## Supporting Information Available

Testing and verification of the simulations; working surfaces of the steady-state limiting current; benchmark; chronoamperogram; concentration profile (PDF)
