# Supplementary material for: Experimental Voltammetry Analyzed Using Artificial Intelligence: Thermodynamics and Kinetics of the Dissociation of Acetic Acid in Aqueous Solution
Source: Anal Chem. 2022 Apr 5;94(15):5901–8. doi: 10.1021/acs.analchem.2c00110 (PMC9082489; doi:10.1021/acs.analchem.2c00110)
Supplement: Supplementary file 1 — ac2c00110_si_001.pdf [file ac2c00110_si_001.pdf]

Supporting Information

Experimental Voltammetry Analyzed Using Artificial Intelligence: Thermodynamics and Kinetics of the Dissociation of Acetic Acid in Aqueous Solution

Haotian Chen[a], Danlei Li[a], Enno Kätelhön[b], Ruiyang Miao[a], Richard G. Compton[a*]

[a] *Department of Chemistry, Physical and Theoretical Chemistry Laboratory, Oxford University, South Parks Road, Oxford OX1 3QZ, Great Britain*

[b] *MHP Management- und IT-Beratung GmbH, Königsallee 49, 71638 Ludwigsburg, Germany*

* Corresponding author.
Email address: Richard.compton@chem.ox.ac.uk (R. G. Compton)

Table of Contents

1 Testing and verification of the simulations 1
2 Working surfaces for the steady-state limiting current....................6
3 Benchmark....................8
4 Chronoamperogram 9
5 Concentration Profile 10
6 References....................12

1 Testing and verification of the simulations

The simulation of the steady state currents described in the text used both expanding space and expanding time grids[1] to reduce computational time and economize on the memory requirement. The expanding space grid can be expressed as:

$$\Delta R_i = R_{i+1} - R_i = \Delta R\ \omega_R^i \#(1)$$

where $0 \leq i \leq (n-1)$, $\Delta \mathrm{R}_i$ is the distance from point i and $i+1$. ΔR is the distance between first two points of the spatial grid and ω_R the spatial expansion factor. The expanding time grid is defined by:

$$\Delta T^k = T^{k+1} - T^k = \Delta T\ \omega_T^i \#(2)$$

where $0 \leq k \leq (m-1)$, ΔT^k is the time interval from point k and $k+1$, ΔT is the time interval between the first two points of the time grid and ω_T is the temporal expansion factor.

For mass conservation during acetic acid reduction to hold, two relationships must be maintained[2-4]:

$$\begin{cases} M_{CH_3COOH}^k + M_{CH_3COO^-}^k = M_1^k \text{ ######## ###} \\ M_{CH_3COOH}^k + M_{H^+}^k + 2 \times M_{H_2}^k = M_2^k \end{cases} \#(3)$$

where m_j^k is the mole of species j at time k. For mass conservation to hold, M_1^k and M_2^k should be constant at all time. The associated error of mass conservation is:

$$\begin{cases} \epsilon_{MC,1} = \dfrac{M_1^f - M_1^0}{M_1^0} \\ \epsilon_{MC,2} = \dfrac{M_2^f - M_2^0}{M_2^0} \end{cases} \#(4)$$

where M_1^f and M_2^f are the values of M_1 and M_2 the end of simulation, and M_1^0 and M_2^0 are those at the start of simulation. $\epsilon_{MC,1}$ and $\epsilon_{MC,2}$ are the fractional errors in mass conservation.

To illustrate the testing of the convergence of, simulations of the dissociative CE reaction of acetic acid with $K_{eq} = 10^{-3}$ and $10^{-5} M$ when $k_f = 10^8\ s^{-1}$, which was the maximum k_f value used in the generation of working surfaces, were assessed. The minimum k_{eq} value used for simulation when $k_f = 10^8\ s^{-1}$ is $10^{-5}\ M$ to ensure $k_b \leq 10^{13}\ M^{-1}s^{-1}$. The bulk concentration of acetic acid was fixed at 10 mM. The steady state current and error of mass conservation with different ΔR,ΔT,ω_R, $and\ \omega_T$ were examined to ensure convergence.

The results are shown in Figure S 1 and Figure S 2. Figure S 1 (a,c,e,g) presents the steady state current at different dR,dT,ω_R $and\ \omega_T$ respectively when $k_f = 10^8 s^{-1}$ and $K_{eq} = 10^{-3} M$ and the currents are converged to -66.85 nA. Figure S 1 (b,d,f,h) shows the steady state current at different dR,dT,ω_R and ω_T respectively when $k_f = 10^8 s^{-1}$ and $K_{eq} = 10^{-5} M$ and the currents are converged to -28.65 nA. Figure S 1 evidences that the simulations are sufficiently converged with the parameters used $\left(dR = 10^{-6}, dT = 10^{-8}, \omega_R = 1.05\ and\ \omega_T = 1.02\right)$.

Figure S 2 and Figure S 3 show similar convergence studies on error of mass conservations. From the figures, all $\epsilon_{MC,1}$ and $\epsilon_{MC,2}$ were all below 0.1%, showing that simulations were sufficiently converged using the parameters mentioned above.

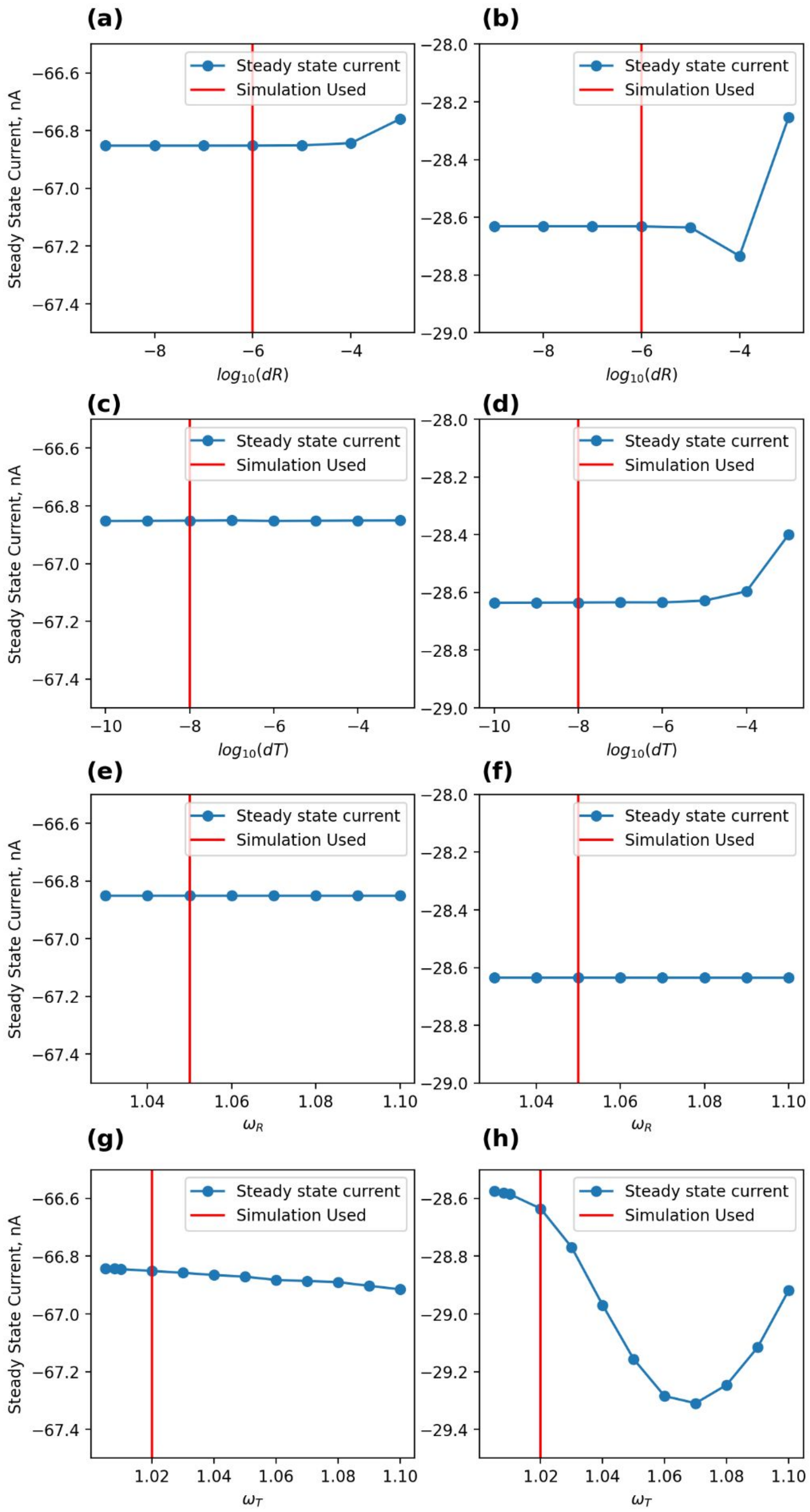

Figure S 1. (a.c.e.g) showed the steady state current at different dR,dT,ω_R and ω_T respectively when $k_f = 10^8 s^{-1}$ and $k_{eq} = 10^{-3}M$. (b.d.f.h) showed the steady state current at different dR,dT,ω_R and ω_T respectively when $k_f = 10^8 s^{-1}$ and $k_{eq} = 10^{-5}M$.

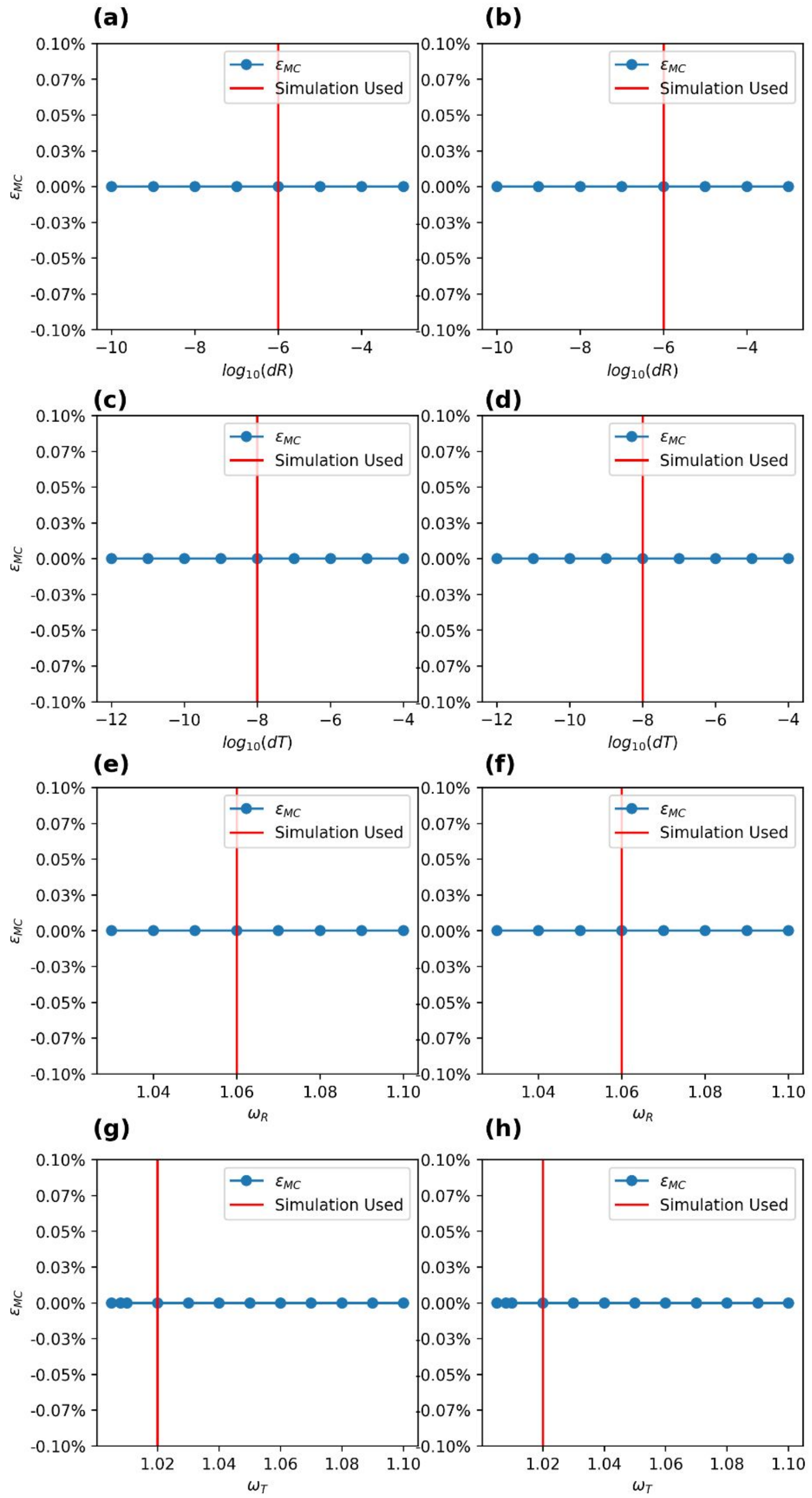

Figure S 2. (a.c.e.g) showed the error of mass conservation ($\epsilon_{MC,1}$) at different dR,dT,ω_R and ω_T respectively when $k_f = 10^8$ s^{-1} and $k_{eq} = 10^{-3}M$. (b.d.f.h) showed the error of mass conservation (ϵ_{MC}) at different dR,dT,ω_R and ω_T respectively when $k_f = 10^8 s^{-1}$ and $k_{eq} = 10^{-5}M$.

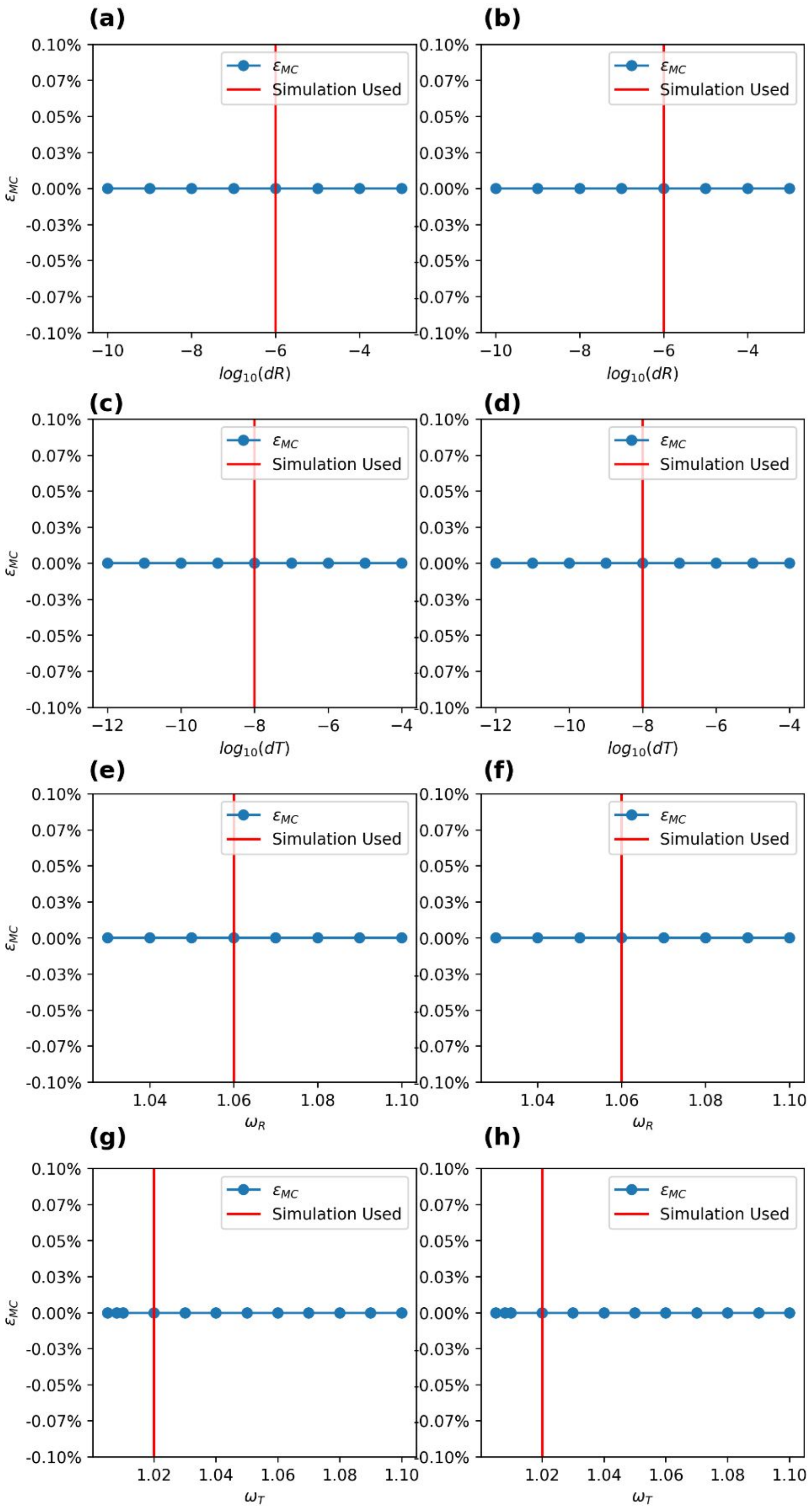

Figure S 3. (a.c.e.g) showed the error of mass conservation ($\epsilon_{MC,2}$) at different dR,dT,ω_R and ω_T respectively when $k_f = 10^8 s^{-1}$ and $k_{eq} = 10^{-3}M$. (b.d.f.h) showed the error of mass conservation (ϵ_{MC}) at different dR,dT,ω_R and ω_T respectively when $k_f = 10^8 s^{-1}$ and $k_{eq} = 10^{-5}M$.

2 Working surfaces for the steady-state limiting current

The working surfaces of steady-state currents for different bulk concentration of acetic acid with various k_f and K_{eq} values were simulated. The working surface when $c^*_{CH_3COOH,total} = 10\ mM$ is shown in the main text, and the working surfaces when $c^*_{CH_3COOH,total} = 20, 40\ and\ 100\ mM$ are shown below.

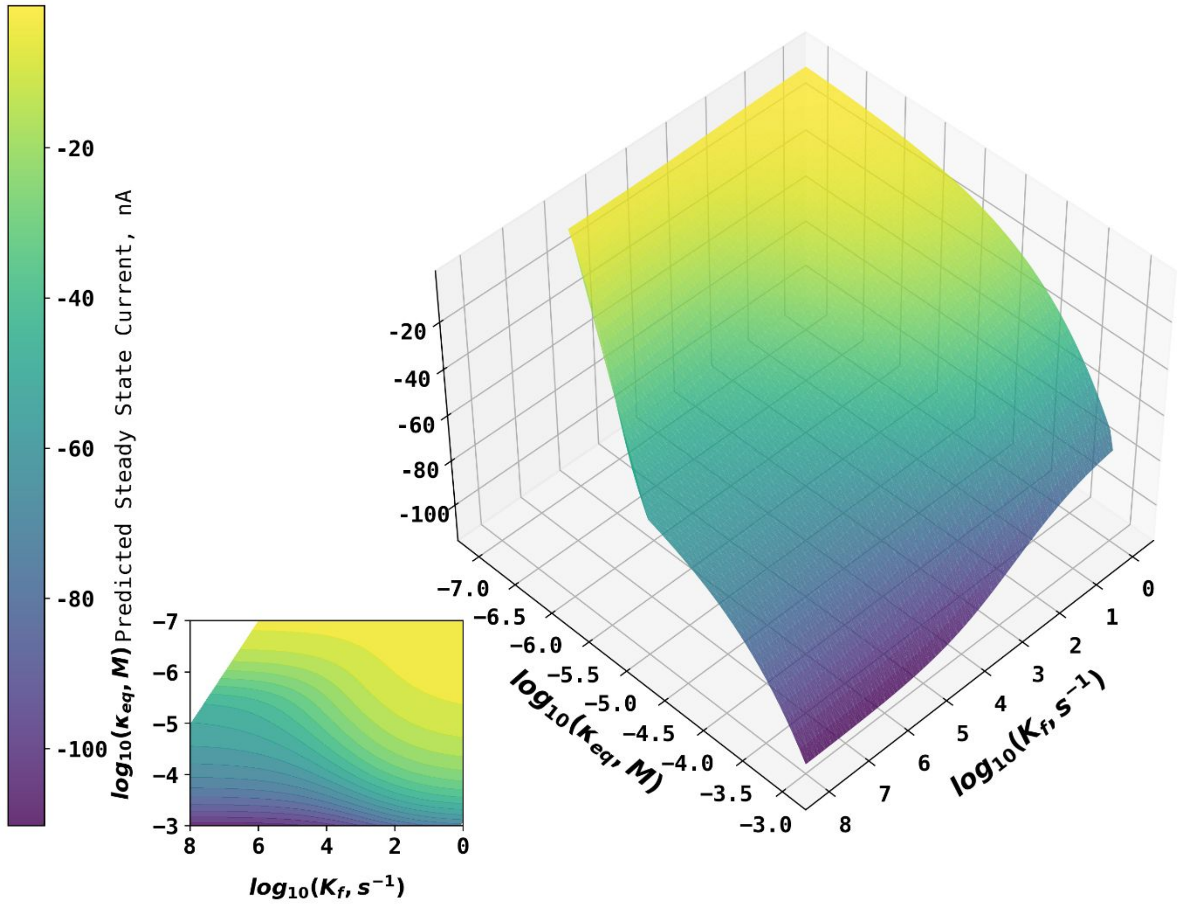

Figure S 4. The working surface showing the steady state currents at different k_f and K_{eq} values for a bulk concentration of acetic acid was 20 mM.

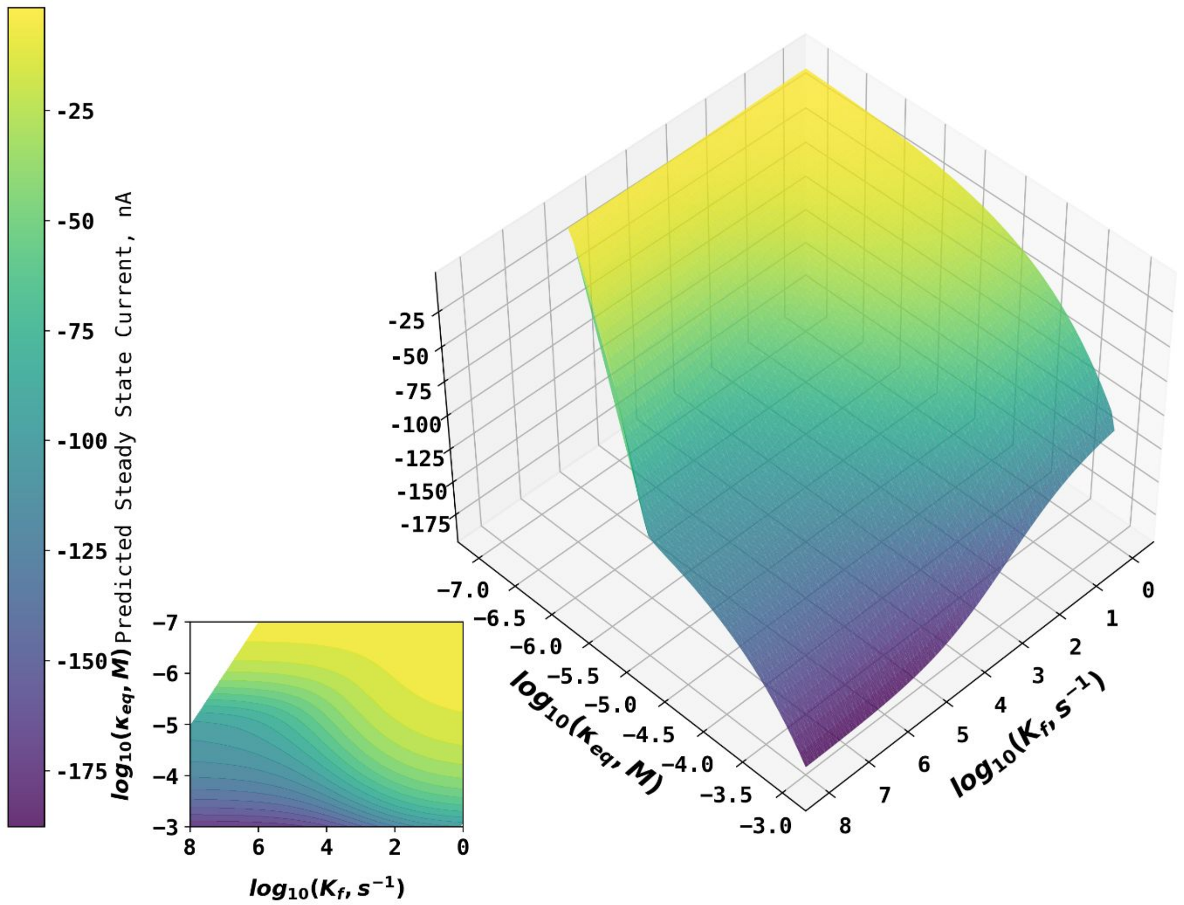

Figure S 5. The working surface showing the steady state currents at different k_f and K_{eq} values for a bulk concentration of acetic acid was 40 mM.

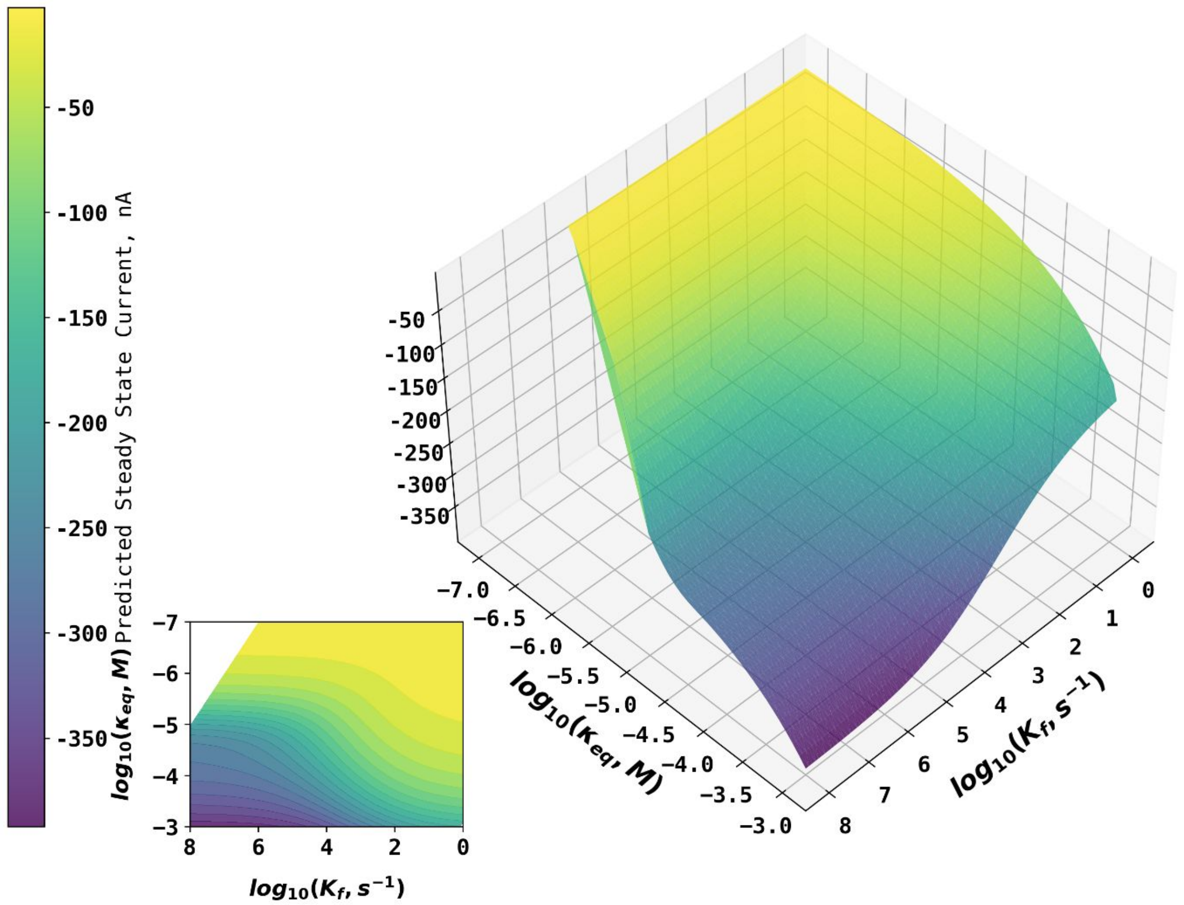

Figure S 6. The working surface showing the steady state currents at different k_f and K_{eq} values for a bulk concentration of acetic acid was 100 mM.

3 Benchmark

To benchmark the performance of the neural network, it was benchmarked with 3rd degree polynomial regression. Using the polynomial regression, 61.2% of prediction of $\log_{10} k_f$within 10% error and 86.7% predictions of $\log_{10} K_{eq}$ within 5% error.

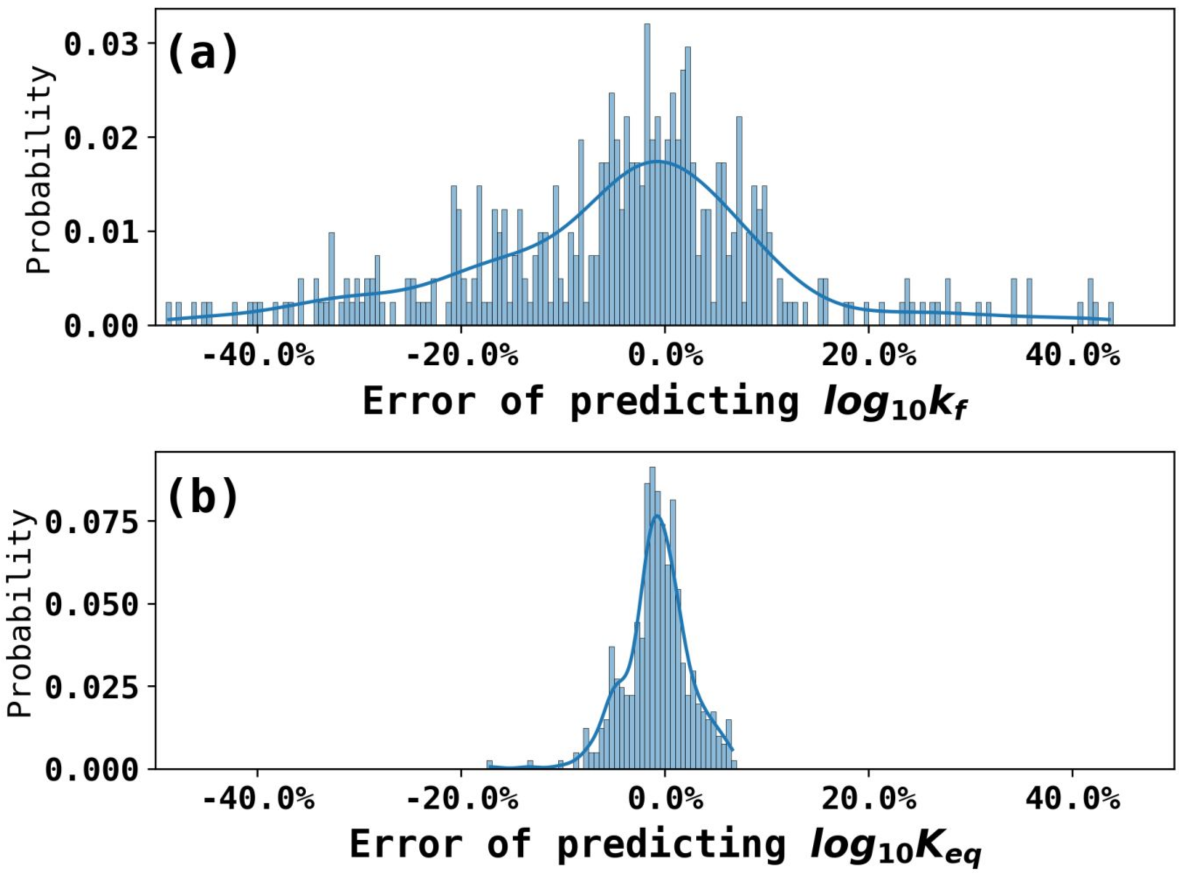

Figure S 7. Error of predicting rate and equilibrium constants from an independent testing dataset composed of simulated steady state currents using 3rd degree polynomial fitting. (a) errors of predicting $log_{10}\, k_f$. 61.2% of predictions of $log_{10}\, k_f$ were within 10% errors; (b) errors of predicting $log_{10}\, K_{eq}$. 86.7% of predictions of $log_{10}\, K_{eq}$ were within 5% errors.

4 Chronoamperogram

Figure S 8 shows the corresponding chronoamperograms recorded for different concentrations of acetic acids recorded for a period of 10 seconds (purple – 10 mM, blue – 20 mM, green – 40 mM and yellow – 100 mM), and they were deemed to have effectively reached steady state behavior after 2 seconds

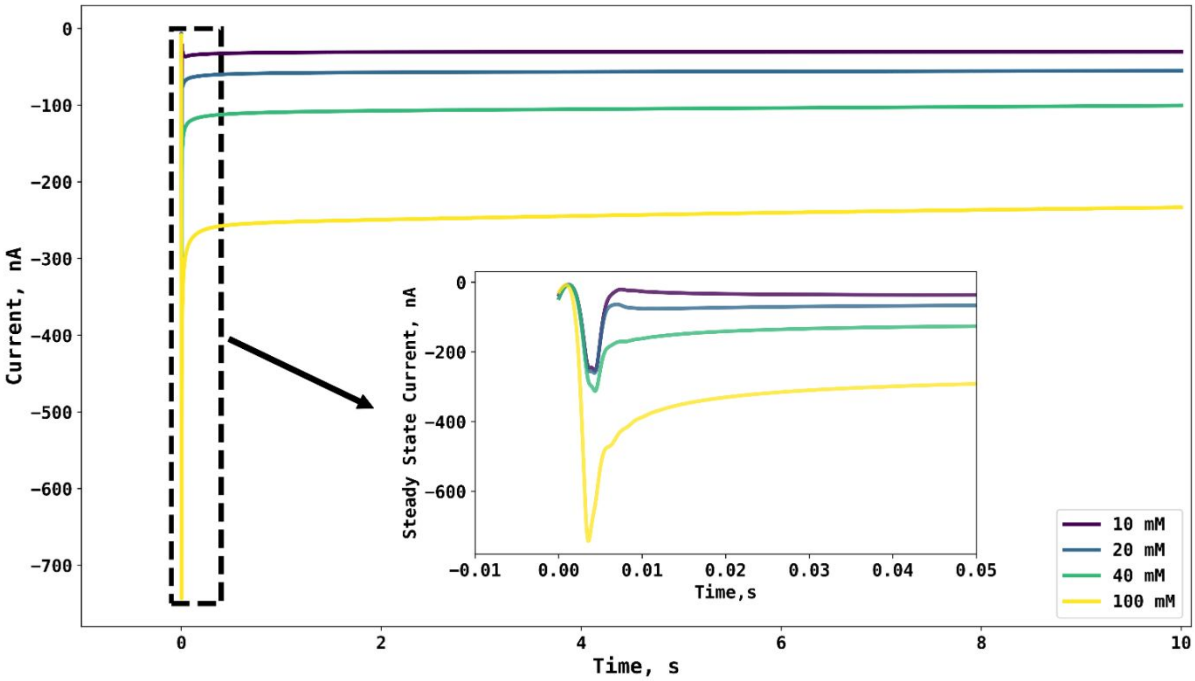

Figure S 8. Chronoamperometry of different concentrations of acetic acid in 0.1M KNO_3 at a Pt microdisc electrode at 298 K. The duration of chronoamperometry scan was 10 seconds and the applied potential was -1.0 V. The inlay figure is a zoom-in view of the chronoamperogram from 0 to 0.05 seconds.

5 Concentration Profile

The steady state concentration profiles of $\mathrm{CH_3COO^-}$, $\mathrm{H^+}$ and $\mathrm{CH_3COOH}$ in in case 1, 2 and 3 are shown in Figure S 9 when (a) $c^*_{\mathrm{CH_3COOH,total}} = 100\ \mathrm{mM}$ and (b) $c^*_{\mathrm{CH_3COOH,total}} = 10\ \mathrm{mM}$. In Figure S 9 and Figure S 10, r/r_e represents the dimensionless spatial coordinate for a spherical electrode, where r and r_e are the distance from the center of an electrode and the radius of the electrode respectively. For example, $r/r_e = 1$ represents the surface of a spherical electrode.

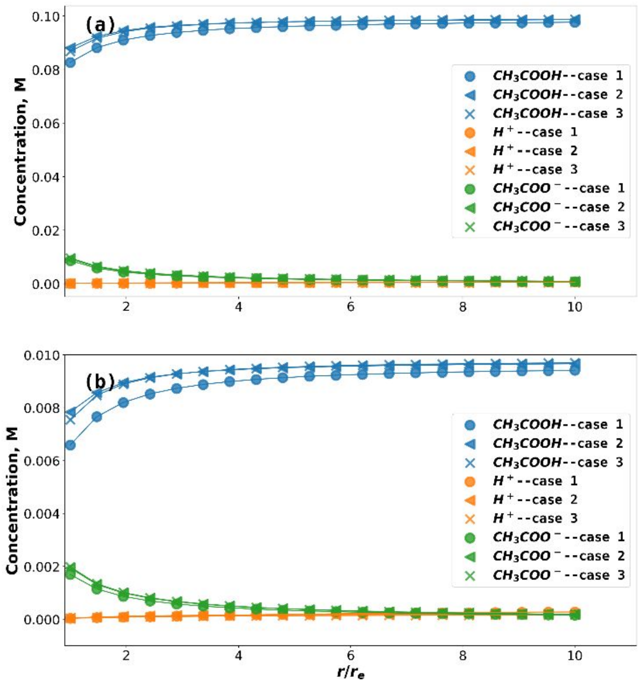

*Figure S 9. Simulated steady state concentration profile for case 1, 2, and 3. (a) $c^*_{CH_3COOH,\,total} = 100\ \ mM$; (b) $c^*_{CH_3COOH,total} = 10\ mM$ (see text and **Error! Reference source not found.**). r/r_e is the dimensionless spatial coordinate for a spherical electrode (see text).*

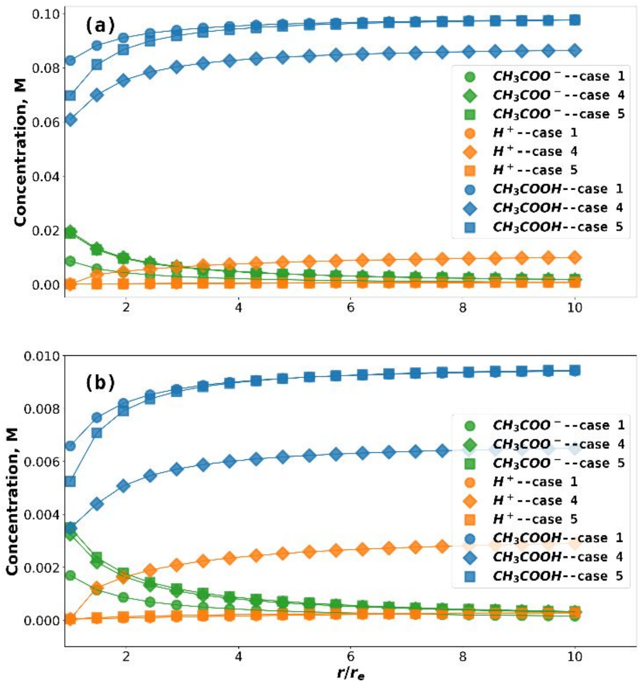

*Figure S 10. Simulated steady state concentration profile for case 1 ,4, and 5. (a) $c^*_{CH_3COOH,\,total} = 100\ \ mM$; (b) $c^*_{CH_3COOH,total} = 10\ mM$(see text and **Error! Reference source not found.**). r/r_e is the dimensionless radial coordinate for a spherical electrode (see text).*

6 References

1. Compton, R. G.; Laborda, E.; Kaetelhoen, E.; Ward, K. R., *Understanding voltammetry: simulation of electrode processes*. 2nd ed.; World Scientific London, 2020.

2. Kätelhön, E.; Compton, R. G., Testing and validating electroanalytical simulations. *Analyst* **2015,** *140* (8), 2592-2598.

3. Kätelhön, E.; Compton, R. G., Correction: Testing and validating electroanalytical simulations. *Analyst* **2015,** *140* (9), 3290-3290.

4. Kätelhön, E.; Compton, R. G., Correction: Testing and validating electroanalytical simulations. *Analyst* **2016,** *141* (3), 1154-1154.
